# Characterization and Transcriptome Analysis of Exosomal and Nonexosomal RNAs in Bovine Adipocytes

**DOI:** 10.3390/ijms21239313

**Published:** 2020-12-07

**Authors:** Binglin Yue, Haiyan Yang, Jiyao Wu, Jian Wang, Wenxiu Ru, Jie Cheng, Yongzheng Huang, Chuzhao Lei, Xianyong Lan, Hong Chen

**Affiliations:** Key Laboratory of Animal Genetics, Breeding and Reproduction of Shaanxi Province, College of Animal Science and Technology, Northwest A&F University, Yangling 712100, Shaanxi, China; yuebinglin@nwafu.edu.cn (B.Y.); yanghaiyan0316@nwafu.edu.cn (H.Y.); wujiyao@nwafu.edu.cn (J.W.); jimwang@nwafu.edu.cn (J.W.); ruwenxiu@nwafu.edu.cn (W.R.); chengjie9412@nwafu.edu.cn (J.C.); hyzsci@nwafu.edu.cn (Y.H.); leichuzhao1118@nwsuaf.edu.cn (C.L.)

**Keywords:** exosomal RNAs, adipocyte, bovine, RNA-seq

## Abstract

Exosomes are endosome-derived extracellular vesicles that allow intercellular communication. However, the biological significance of adipocyte exosomal RNAs remains unclear. To determine the role of RNAs from bovine adipocytes and exosomes in bovine adipogenesis, exosomal and nonexosomal RNAs were extracted from three bovine primary white adipocyte samples and then profiles were generated using DNBSEQ/BGISEQ-500 technology. The RNAome of adipocytes consisted of 12,082 mRNAs, 8589 lncRNAs, and 378 miRNAs for a higher complexity that that detected in exosomes, with 1083 mRNAs, 105 lncRNAs, and 48 miRNAs. Exosomal miRNA-mRNA and lncRNA–miRNA–mRNA networks were constructed and enrichment analysis was performed to predict functional roles and regulatory mechanisms. Our study provides the first characterization of RNAs from bovine adipocyte and exosomes. The findings reveal that some RNAs are specifically packaged in adipocyte-derived exosomes, potentially enabling crosstalk between adipocytes and/or other cells that is mediated by exosomes. Our results greatly expand our understanding of exosomal RNAs from bovine adipocytes, and provide a reference for future functional investigations of adipocyte exosomal RNAs under normal physiological conditions.

## 1. Introduction

In mammals, adipose tissues serve as the largest energy storage organs, playing crucial roles in whole body energy homeostasis by secreting multiple active substances, such as adipokines [1]. Adipose tissues are composed of mostly mature adipocytes, as well as stromal cells such as preadipocytes, fibroblasts, endothelial cells, and immune cells [2]. In general, there are two main kinds of adipocytes: white adipocytes and brown adipocytes. White adipocytes can recycle and release fatty acids through lipogenesis and lipolysis, respectively, and brown adipocytes consume glucose and lipids to modulate thermal homeostasis [3]. Alterations in the mass of white adipose tissue (WAT) result from changes in white adipocyte number and/or size, in processes known as hyperplasia and hypertrophy, respectively. Adipocyte hyperplasia is related to a complex crosstalk between the proliferation and differentiation of preadipocytes, and this crosstalk is tightly regulated by both cell cycle regulators and differentiating factors. The synthesis or uptake of fatty acids leads to the accumulation of lipids in adipocytes, resulting in hypertrophy [4]. Beyond energy metabolism, the function of WAT as a dynamic secretory organ with pleiotropic functions was recently demonstrated, as diverse factors released by adipocytes can directly act on cells (autocrine) or interact with neighboring (paracrine) or distant (endocrine) cells to affect a variety of biological and pathological processes [5].

Accumulating evidence suggests that cells can secret exosomes, a kind of nanoscale membrane vesicles (<100 nm) that protect contents such as nucleic acids, proteins, and lipids from degradation and facilitate intercellular transmission, altering behaviors of recipient cells [6,7]. Exosomes are formed by inward budding of the plasma membrane, and recipient cells acquire exosomes through a range of endocytic pathways, including phagocytosis, clathrin-dependent endocytosis, and caveolin-mediated uptake [8,9]. Exosome content is highly heterogenous, depending on the origin and physiopathologic conditions of donor cells [10]. There is growing interest in the discovery and characterization of exosomal RNAs, including coding RNAs and noncoding RNA species such as miRNAs and lncRNAs, as some of these RNAs have specific functions and are selectively enclosed by exosomes in donor cells [11,12]. Adipocytes can also secrete exosomes, and there is growing interest in studying the function of adipocyte exosomal RNAs, however, the distribution characteristics and comprehensive expression profile of RNAs from adipocyte-derived exosomes remain poorly understood [13].

In this study, we systematically characterized exosomal and nonexosomal RNA profiles during the proliferation of bovine primary white adipocytes using deep sequencing technology. Based on the obtained RNA profiles, the potential sources of exosomal RNAs were determined and the potential biological functions were predicted using bioinformatics analysis. These findings provide insight into the physiological functions of bovine adipocyte-derived exosomal RNAs, and these results deepen our understanding of the biological significance of these RNAs in adipogenesis in mammals.

## 2. Results

### 2.1. Identification of Adipocyte Exosomes in Cell Culture Medium

**Figure 1:**
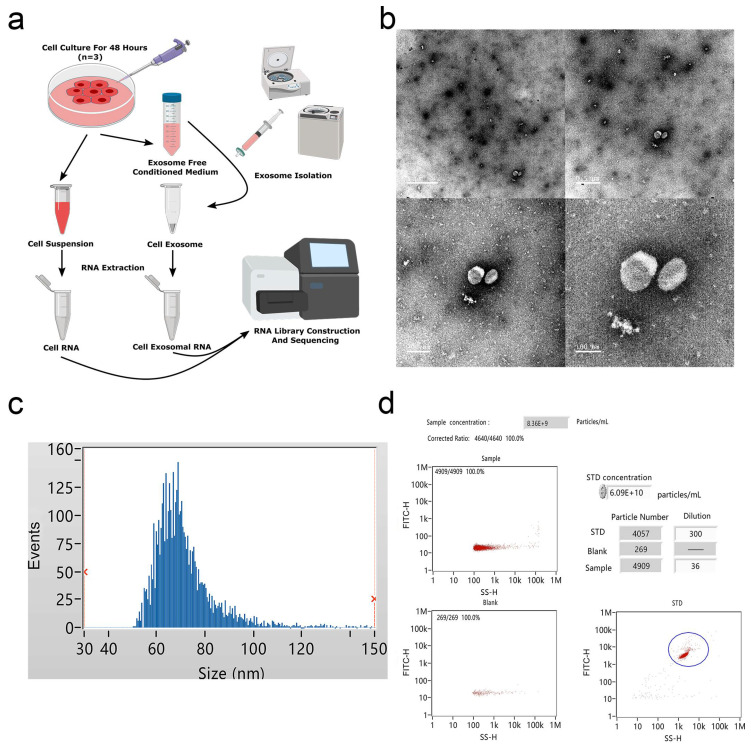
Identification of bovine adipocytes-derived exosomes. (**a**) Exosome collection procedure from bovine adipocytes conditioned medium. (**b**) Transmission electron microscope (TEM) images of exosome isolations using ultracentrifugation. Scale bar = 1 μm, 0.5 μm, 200 nm, and 100 nm. (**c**) The size distribution of the purified exosomes was detected using Flow NanoAnalyzer. (**d**) Analysis of exosomal concentrations corresponding to the data in (**c**).

To profile exosomal RNAs, we extracted exosomes from cultured medium of bovine adipocytes and prepared RNA-seq libraries for high-throughput sequencing (Figure 1a). TEM examination of the samples revealed exosome pellets that were spherical in structures, with diameters of nearly 100 nm (Figure 1b). NTA measurement indicated that most particles were between 60 and 80 nm in size (Figure 1c), and the particle concentration at a 5-fold dilution was approximately 8 × 10^9^ particles/mL (Figure 1d), consistent with typical exosome morphology. As shown in Supplementary Materials Figure S1, Western blot analysis determined the expression of exosome surface biomarkers (CD63 and Alix).

### 2.2. Overview of RNA Sequencing Data

**Figure 2:**
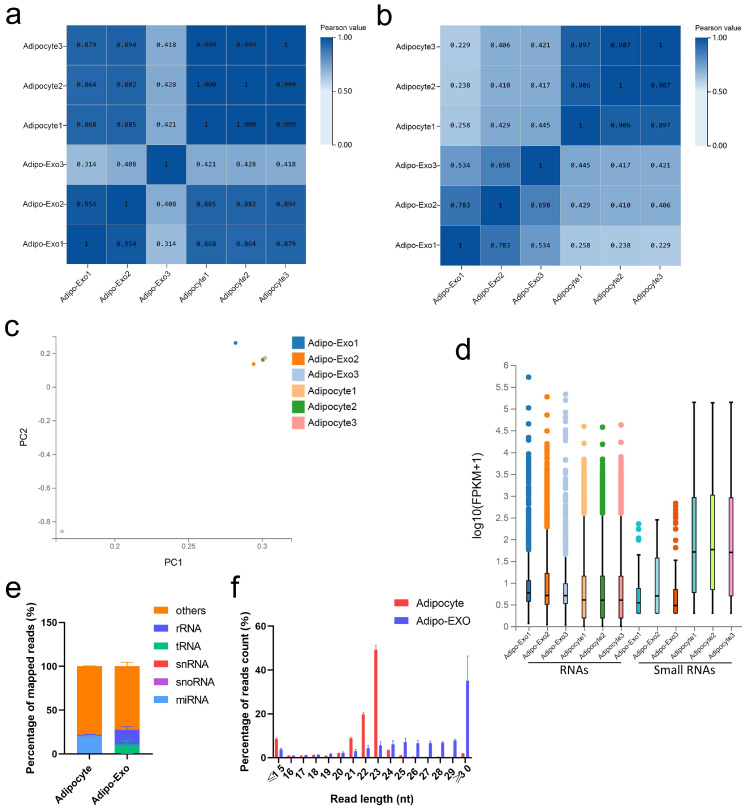
Analysis of the sequencing data. (**a**,**b**) Heat maps showing Pearson correlations of RNA (**a**) and small RNA (**b**) from adipocyte-derived exosomes and adipocytes. (**c**) Principal component analysis (PCA) of all normalized RNA sequencing data. (**d**) Box plot of detected RNAs. (**e**) Percentage of different classes of small RNAs. (**f**) Length and frequency distributions of total small RNAs. Error bars indicate the SEM.

DNBSEQ/BGISEQ-500 technology was applied to systematically identify exosomal and nonexosomal RNAs from bovine adipocyte-derived exosomes and bovine adipocytes, respectively. A total of 12 sequencing libraries were obtained (including six small RNA libraries). High-throughput sequencing produced a mean of 110.8 million reads per sample, and small RNA sequencing showed an average of about 21.6 million tags per library. The q20 values of clean data for these two libraries were >97% and > 99%, respectively, indicating the high quality of sequencing results (Tables S1–S3). Except for an outlier (adipo-exo3), the fractions of adipocyte samples were more similar to each other than to the samples of the adipo-exosome fractions and vice-versa (Figure 2a,b). Principle component analysis (PCA) was used to analyze the distribution and uniformity of the independent samples. Similar RNA profiles were obtained for adipocytes and adipo-exosomes (except for adipo-exo3) (Figure 2c). A boxplot was used to evaluate the overall quality of the data, including the distribution of RNA expression levels for each sample and the degree of dispersion of the data distribution (Figure 2d). From these data, we see that there is a relatively low abundance of small RNAs in the exosomes. Additionally, our results showed 20.3% annotated miRNAs in adipocytes and 0.01% annotated miRNAs in adipo-exosomes (Figure 2e, Table S4). As shown in Figure 2e, the percentages of snRNAs and snoRNAs were low in both adipocytes and adipo-exosomes, with abundant rRNAs and tRNAs in adipo-exosomes. We also analyzed the length and frequency distribution of total small RNAs and found that most RNAs in adipocytes were 20–25 nt in length, while longer RNAs were found in adipo-exosomes, with a peak in length ≥ 30 (Figure 2f, Table S5). These results suggest there is a mechanism to control the sort of RNA put into exosomes.

### 2.3. RNA Profiling in Adipocytes and Released Exosomes

**Figure 3:**
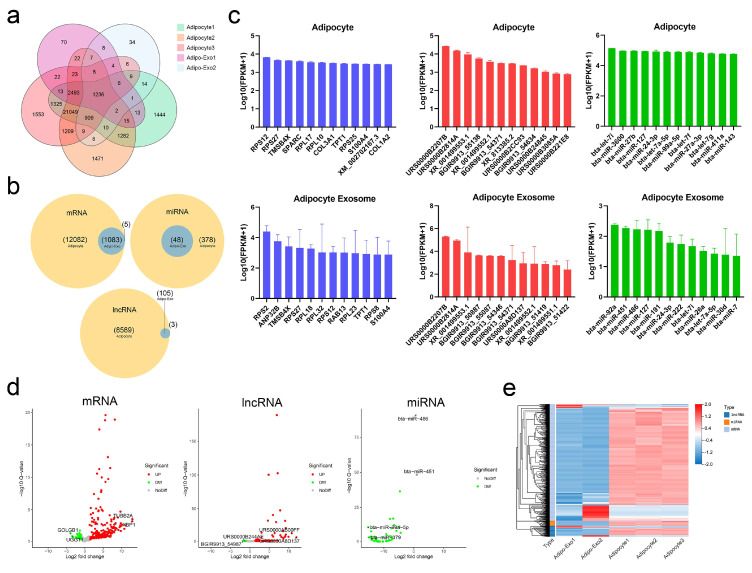
Profiles of exosomal and nonexosomal RNAs. (**a**,**b**) Venn diagrams showing the profile of total RNAs (**a**) and three types of RNA (**b**) overlaps between adipocytes and released exosomes. (**c**) Histograms of the 12 most abundant mRNAs (blue), lncRNAs (red), and miRNAs (green) from adipocytes and released exosomes. (**d**) Volcano maps of differentially expressed exosomal mRNAs, lncRNAs, and miRNAs according to the criteria of fold change ≥ 2 between parental adipocytes and exosomes. (**e**) Heatmap of differentially expressed RNAs between parental adipocytes and exosomes.

In our analysis, adipo-exo3 was removed as an outlier (as described above) and we focused on RNAs such as mRNAs, lncRNAs, and miRNAs. We compared the RNA sets obtained from adipocytes and from released exosomes, and observed 1236 RNAs (Figure 3a) that were commonly expressed in adipocytes and released exosomes, including 1083 mRNAs, 105 lncRNAs, and 48 miRNAs (Figure 3b). A total of 21,049 RNAs (12,082 mRNAs, 8589 lncRNAs, and 378 miRNAs) were only detected in adipocytes and were not be expressed in released exosomes, indicating adipocytes exported only a subpopulation of RNAs into exosomes and that the process of RNA packing into exosomes is selective (Figure 3a,b). In addition, five mRNAs (EFL1, CPS1, PCM1, SH3D19, and XM_015468604.1) and three lncRNAs (BGIR9913_49345, BGIR9913_54344, and URS0000B2F7C9) were detected in exosomes irrespective of cellular origin, intimating potential different mechanisms in sorting of RNA cargoes. As shown in Figure 3c, the 12 most abundant mRNAs, lncRNAs, and miRNAs were separately filtered out from adipocytes and released exosomes. Comparison of these filtered RNAs showed adipocytes and adipo-exosomes exhibited similar profiles of abundant RNAs, with some of the same mRNAs (TMSB4X, RPS27, RPS12, TPT1, and S100A4), lncRNAs (URS0000B2207B, URS0000B2814A, XR_001499553.1, BGIR9913_54371, and XR_001499552.1), and miRNAs (bta-miR-127, bta-miR-24-3p, bta-let-7i, and bta-let-7a-5p) (Figure 3c). Applying the criteria of fold change ≥2, differential expressed RNAs between parental adipocytes and exosomes were identified, including a total of 498 mRNAs (386 upregulated and 112 downregulated), 68 lncRNAs (62 upregulated and six downregulated), and 46 miRNAs (all downregulated). Volcano plots and hierarchical clustering consistently showed variation of lncRNA, mRNA, and miRNA expression between parental adipocytes and exosomes (Figure 3d,e). All of these data revealed altered RNA profiling between adipocytes and released exosomes and indicated the selective assembly of RNAs in exosomes secreted from adipocytes rather than a random process.

### 2.4. Functional Enrichment Analysis of Exosomal mRNAs

**Figure 4:**
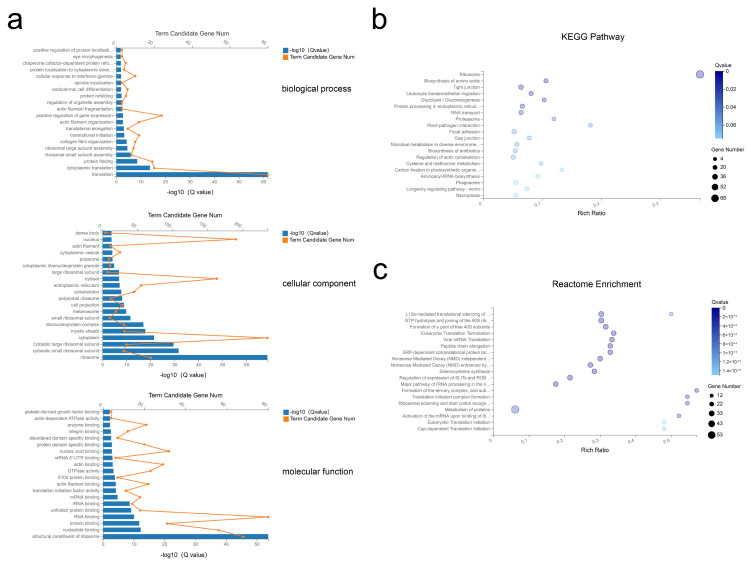
GO, KEGG, and reactome enrichment analysis of exosomal mRNAs. (**a**) The 20 most enriched GO terms including biological process, cellular component, and molecular function. (**b**,**c**) Advanced bubble chart of KEGG pathway annotation (**b**) and reactome enrichment analysis (**c**) with 20 highest enrichment scores.

Next, the top 500 abundant exosomal mRNAs were filtered out and functional enrichment was assessed. Gene ontologies (GO) analysis revealed enrichment of 49 Biological Process (BP), 84 Cellular Component (CC), and 57 Molecular Function (MF) terms with Q < 0.05 (Tables S6–S8). For the most significantly enriched components, BP terms included translation, cytoplasmic translation, and protein folding; CC terms of ribosome, cytosolic small ribosomal subunit, and cytosolic large ribosomal subunit; MF terms included structural constituents of ribosome, nucleotide binding, and protein binding (Figure 4a). Applying the criteria of *p*-value < 0.05, analysis revealed 34 Kyoto Encyclopedia of Genes and Genomes (KEGG) pathways were enriched (Table S9), with many pathways related to the ribosome, biosynthesis of amino acids, and protein processing in endoplasmic reticulum (Figure 4b). Further, reactome pathway enrichment analysis showed significantly affected pathways included formation of a pool of free 40S subunits, eukaryotic translation termination, and peptide chain elongation (Figure 4c, Table S10). Overall, these results suggested that the selected exosomal mRNAs function in the process of protein synthesis.

### 2.5. Construction and Analysis of the miRNA–mRNA Network

**Figure 5:**
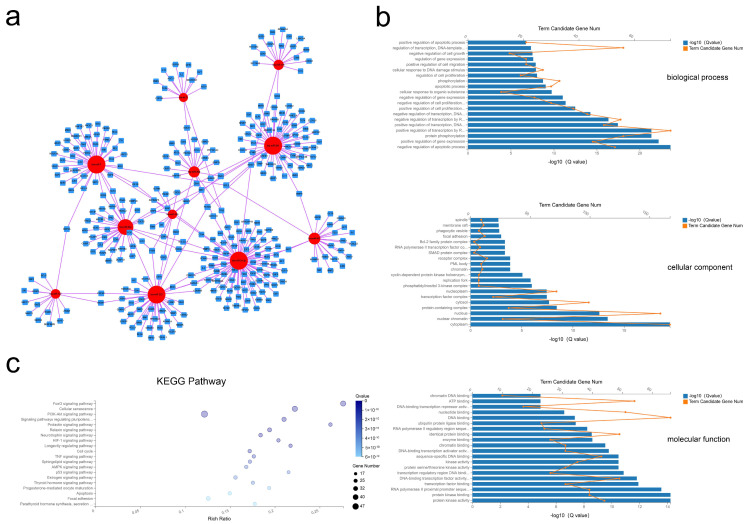
Exosomal miRNA–mRNA network construction and analysis. (**a**) Global view of the exosomal miRNA–mRNA network, including 11 miRNAs (red circles), and 328 mRNAs (blue squares). (**b**) The top 20 GO terms including biological process, cellular component, and molecular function. (**c**) Advanced bubble chart of the 20 most enriched KEGG pathways.

After processing, 11 miRNAs and 328 mRNAs were identified and used to assemble the miRNA–mRNA network (Figure 5a). In this analysis, each node represents an RNA, and each edge represents an interaction between two RNAs. The node degree typifies the number of edges linked to a node, where the higher the degree, the bigger the size, indicating biological functions. GO analysis revealed 633 BP, 45 CC, and 115 MF terms with Q < 0.05 (Tables S11–S13). By this analysis the most significant terms were BP terms including negative regulation of apoptotic process, positive regulation of gene expression, and protein phosphorylation; CC terms of cytoplasm, nuclear chromatin, and nucleus; and MF terms of protein kinase activity, protein kinase binding, and RNA polymerase II proximal promoter sequence-specific DNA binding (Figure 5b). Applying the criteria of Q-value < 0.05, KEGG analysis revealed enrichment of 88 pathways (Table S14), with many of these pathways related to the FoxO signaling pathway, the PI3K-Akt signaling pathway, and the cell cycle (Figure 5c). These results suggested that selective exosomal miRNAs act in processes of cell proliferation and apoptosis.

### 2.6. Coexpression of the lncRNA–miRNA–mRNA Network and Functional Prediction

**Figure 6:**
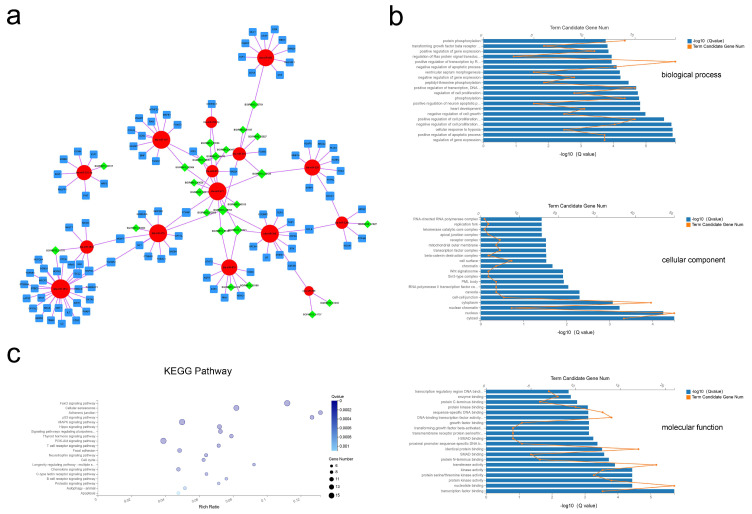
Exosomal lncRNA–miRNA–mRNA network construction and analysis. (**a**) Global view of the exosomal lncRNA–miRNA–mRNA network, including 24 lncRNAs (green diamonds), 15 miRNAs (red circles), and 102 mRNAs (blue squares). (**b**) The 20 most enriched GO terms including biological process, cellular component, and molecular function. (**c**) Advanced bubble chart of the 20 most enriched KEGG pathways.

As shown in Figure 6a, 24 lncRNA, 15 miRNA, and 98 mRNA composed the lncRNA–miRNA–mRNA network (Figure 6a). The results of the GO analysis revealed 493 BP, 26 CC, and 89 MF terms with Q < 0.05 (Tables S15–S17). The most significant enrichment was for BP terms of regulation of gene expression, positive regulation of apoptotic process, and regulation of cell proliferation; CC terms including cytosol, nucleus, and nuclear chromatin; MF terms of transcription factor binding, nucleotide binding, and protein kinase activity (Figure 6b). Applying the criteria of Q-value < 0.05, 50 KEGG pathways were enriched (Table S18), with many of these pathways related to the FoxO signaling pathway, cellular senescence, and the p53 signaling pathway (Figure 5c). These data show that bovine adipocytes released exosomal lncRNAs that are predicted to regulate cell-based proliferation and apoptosis.

## 3. Discussion

Since the initial identification of leptin and adiponectin, WAT has been recognized as a dynamic secretory organ with pleiotropic functions beyond energy metabolism [14]. WAT includes a considerable variety of cell types, including preadipocytes, fibroblasts, immune cells, and endothelial cells [15,16,17,18]. Cells secrete diverse factors and adjacent cellular communication is mediated by gap junctions or tunneling nanotubes [19]. The recent characterization of exosomes and their carried contents has led to an extension of our understanding of intercellular communication. Exosomes provide a natural carrier system that can transfer carried contents such as proteins, nucleic acids, and lipids between donor and receiver cells, acting to protect loaded cargoes from being eliminated by the mononuclear phagocyte system and providing a flexible mechanism of intercellular transfer [20]. Several reports have characterized the potential RNA contents of exosomes, and exosomal lncRNA and miRNA have demonstrated functions in recipient cells. However, only preliminary characterization and analysis of exosomes and carried RNAs in adipocytes have been performed. In this work, we isolated and verified bovine white adipocyte-derived exosomes; comprehensively profiled the mRNA, lncRNA, and miRNA transcriptomes of exosomes using RNA-seq; ultimately evaluated the molecular relatedness of these RNAs and analyzed their putative functions.

Our data provided the first insight into differences between bovine adipocytes and released exosomes, and results supported the hypothesis of directed RNA export in exosomes. Both Pearson correlation analysis and PCA showed highly correlated RNA profiles between adipocytes and adipo-exosomes, except for one adipo-exo3, so this one was removed as an outlier in our subsequent analysis. The RNA sequencing revealed generally higher expression of exosomal mRNAs and lncRNAs than that of cellular mRNAs and lncRNAs, and lower miRNA expression levels in exosomes compared to that in adipocytes, indicating differential sorting of various RNAs into exosomes. Several recent studies have reported mechanisms involved in the selective targeting of RNAs into exosomes. For example, some motifs enriched in mRNA 3′-UTR may potentially act as cis-acting elements to promote targeting of mRNAs into exosomes [21], and targeting lncRNA to exosomes occurs via a specific protein hnRNPA2B1, which characteristically binds to the 5′ sequence of lncARSR to guide lncRNA sorting into exosomes [22]. The packaging of miRNAs into exosomes can be mediated by the neural sphingomyelinase 2 (nSMase2)-dependent pathway [23] and 3′ end uridylation [24], as well as by RNA-binding proteins such as Ago2 [25], heterogeneous nuclear ribonucleoproteins (hnRNP) family protein [26], and Y-box protein 1 (YBX1) [27]. Specific lncRNA–RBP complexes can capture special miRNAs and sort them into exosomes [28]. As the most common fragments ranging in size from 19 to 25 nt, miRNAs make up the largest fraction of RNA in cells, however, only 0.01% miRNAs were detected in exosomes. Interestingly, abundant rRNAs and tRNAs were found in adipocytes and released exosomes. Similar to previous reports of exosomal RNA enrichment [29,30], we found that exosomes were enriched with 5S ribosomal RNA in the absence of visible 18S and 28S rRNA peaks on exosomal RNA profiles (Figure S2). Another abundant RNA species, tRNAs represented over 50% of the total small RNAs in adipose-derived exosomes. Although it is unknown whether these exosomal rRNAs and tRNAs have a specific function, some limited evidence has suggested the presence of fragmented rRNA and tRNA in exosome, which may represent novel RNA types with potential regulatory functions [30,31]. The enrichment of small RNAs also exhibited a similar size distribution as that previously reported in bovine sera and exosomes with a ≥30 peak observed in adipo-exosomes; this peak may correspond to processed small RNAs acting as a novel form of signaling molecules [32]. However, further investigation is required to determine whether and how these different classes of exosomal RNA influence recipient cells.

In this study, a total of 1236 RNAs were normally expressed in adipocytes and released exosomes, including 1083 mRNAs, 105 lncRNAs, and 48 miRNAs. Although fewer in number, these exosomal RNA profiles were closely correlated with those obtained previously for native cells. This is consistent with a wide array of studies suggesting that cell-derived exosomal RNA profiles closely reflect RNA profiles in parental cells, and these exosomal RNAs may transfer intercellular signals to affect critical biological functions [33,34]. First detected in murine and human mast cell lines (MC/9 and HMC-1), exosomal mRNAs have been described as important regulators of cell behavior, with approximately 1300 mRNAs detected from MC/9 exosomes by microarray analysis and associated with ontologies such as RNA post-transcriptional modification and protein synthesis. Interestingly, the transfer of murine exosomal mRNA to the HMC-1 generates the output of new murine proteins in the HMC-1, indicating that exosomal mRNAs can be translated into proteins in recipient cells [35]. We observed enrichment in exosomes for mRNAs that encode ribosomal proteins in the small (RPS) or large (RPL) subunits, such as RPS2, RPS12, RPS27, RPL18, RPL23, and RPL32, and functional annotations of exosomal mRNAs have been consistently linked to the biosynthesis of amino acids and protein processing and modification [35,36]. Exosomal mRNAs released from adipocytes can be transmitted to recipient cells, where they can facilitate the protein expression programs of recipient cells. Jenjaroenpun et al. also hypothesized that exosomal mRNAs can be translated into proteins that support ribosomal function after being delivered to recipient cells, facilitating the efficacious translation of other exosomal mRNAs in a cellular domain where exosomal content is released [30].

Our results showed only a small number of miRNAs in adipocyte exosomes, but the few miRNAs detected may be important in adipocyte biology. Specifically, expression of miR-143, miR-15b, and let-7 subtypes was expressed in both adipocytes and released exosomes. Ectopic expression of miR-143 in preadipocytes accelerates the rate of adipocyte formation and acts in adipocyte differentiation by targeting Erk5 [37]. In porcine preadipocytes, the inhibition of FOXO1 expression by miR-15b indicates a positive effect on adipogenesis [38]. The let-7 miRNA modulates the larva to adult transition in *caenorhabditis elegans*, and a study on 3T3-L1 cells supports a role for let-7 in the transition to terminal differentiation during adipogenesis [39,40]. With high conservation across species and wide regulatory roles in target gene expression, these miRNAs may directly influence cell functions once released into other adipocytes. Interestingly, there is no report on the effects on adipogenesis by these abundant exosomal miRNAs, but there is potential for significant regulation of surrounding cells. As major exosomal signals and effectors, a broad range of adipocyte-derived miRNAs can transfer into recipient cells via exosomes, resulting in the suppression of mRNA targets. For example, miR-27a can repress adipogenesis by targeting PPARγ, and recent studies demonstrated that adipocyte-derived exosomal miR-27a could be assimilated by myocytes triggering insulin resistance in skeletal muscle through repression of PPARγ [41]. Pan et al. identified adipocyte-secreted miR-34a as a key paracrine mediator that suppresses M2 polarization by repressing KLF4 in adipose-resident macrophages [13]. Another miRNA, miR-23a/b is delivered from adipocytes to cancer cells, and miR-23a/b was shown to act to modulate HCC cells by repressing the expression of VHL/HIF-1α [42]. The functional exosomal miRNAs described above were all found in bovine adipocyte-released exosomes, which increase the credibility of our sequencing results to a certain degree. Similarly, the abundant exosomal miR-451 detected in this study has also been reported as a highly exported exosomal miRNA in many different cell types, where it has been shown to be processed by the Ago2 protein rather than RNase III Dicer into mature miRNA duplexes [43,44]. To identify functions of abundant exosomal miRNAs, GO-based target prediction was performed and revealed potential roles for these miRNAs in apoptotic process, regulation of cell proliferation, and protein phosphorylation, with significant enrichment of pathways related to FOX and PI3K-AKT3 signaling. These predictions suggest possible activities of these exosomal miRNAs in cell proliferation and apoptosis, however detection in exosomes does not necessarily mean that all these miRNAs are transferred into other cells, so this requires further investigation.

As mRNA-like transcripts over 200 nucleotides length with abundant binding sites for miRNA, lncRNAs competitively interact with miRNA to repress mRNA targets expression, in a mechanism known as competing endogenous RNA (ceRNA) [45]. With low conservation across species, almost all of our identified lncRNAs have not been reported in previous studies. Accordingly, we constructed an exosomal lncRNA–miRNA–mRNA network for exosomal lncRNAs annotation. The ceRNA network has been shown to regulate a variety of cellular processes in adipocytes. For example, lncRNA ADNCR can inhibit adipocyte differentiation via sponging miR-204, indirectly augmenting gene expression of SIRT1, which can inhibit adipocyte formation [46]; GAS5 can compete for miR-18a/CTGF to suppress adipogenic differentiation of mesenchymal stromal cells (MSCs) [47]; RP11-142A22.4/miR-587/Wnt5β axis is a potential pathway to promote adipogenesis through the ceRNA mechanism [48]. However, there are few ceRNA studies about exosomal lncRNAs. The network analysis results indicated that BGIR9913_50763, BGIR9913_55051, BGIR9913_51021, and BGIR9913_52429 are crucial components of this network, with links to the greatest number of miRNAs. Furthermore, functional enrichment analysis revealed significant enrichment of these exosomal lncRNAs in cell proliferation and apoptosis-related biological processes, including the FOX and p53 signaling pathways, suggesting their importance in cell growth and development.

As a natural cell product, exosomes transport functional RNAs between cells, avoiding their degradation by phagocytes or lysosomes [49]. Hence, exosomes are logically considered as key contributors to mechanisms that adipocytes can employ to influence other cell systems. In this study, we investigated the RNA transcriptome of bovine adipocytes and their released exosomes, using deep sequencing technology, expanding our understanding of the physiological functions and mechanisms of adipocyte exosomal RNAs. To our knowledge, this is the first systemic study of adipocyte exosomal RNAs in bovine, and the results of this work should provide a reference for future functional studies on adipocyte exosomes under normal physiological conditions. However, there are still some limitations of this work that should be acknowledged. Given the current limitations of exosome separation techniques, it is difficult to completely discriminate exosomes and specific subtypes from other vesicle types, resulting in the heterogeneity of RNAs in exosomes [9]. Therefore, our exosomal RNAs in the libraries may be merely subsets of all RNAs isolated in this experiment. Overall, improvements in the stringent purification of exosomes are required. Similarly, it is difficult to separate high-purity adipocytes from WAT, raising the possibility that exosomes from fibroblasts, endothelial cells, or immune cells may contaminate our exosome isolation. In addition, reads from RNA-seq are calculated based on signal intensities, which are not always accurate due to technical limitations, so biological verification should be performed to validate the results of sequencing. Recently, coculture systems were used to reproduce cellular interactions, with exosomal RNAs produced by overexpression of exosomes [50,51]. However, it remains unclear whether these overexpressed exosomes can produce functional RNAs that can be transferred in native biological settings, and the slight induction of exosomal RNAs observed in target cells may not be sufficient to produce significant effects. Furthermore, with some common targets RNAs in the exosomes may collaborate to induce target cell phenotypes. These issues will need to be addressed in future work.

## 4. Materials and Methods

### 4.1. Ethics Statement, Sample Collection, and Cell Culture

All animal experiments were conducted according to the Regulation for the Administration of Affairs Concerning Experimental Animals (Shaanxi, China, 2017), and our study was approved by the Northwest A&F University Institutional Animal Care and Use Committee (November 2017). Adipose tissues were acquired from Qinchuan cattle at the embryonic stage (90 days), and these samples were provided by Shannxi Kingbull Animal Husbandry (Baoji, China). Adipocytes were extracted from bovine adipose tissues as previously detailed [52,53], and were then seeded into 100-mm plates and cultured at 37 °C with 5% CO_2_ until they reached 90% confluence using DMEM medium (20% exosomes-depleted fetal bovine serum with 1% penicillin-streptomycin). Three biological replicates were performed for adipocyte culture.

### 4.2. Exosome Isolation

The conditioned medium was collected in a fresh 50 mL tube and centrifuged at 800 rpm for 10 min followed by 12,000 rpm for 30 min at 4 °C to remove debris. After centrifugation, the supernatant was filtered using a 0.22-µm filter (Millipore, Burlington, MA, USA) to eliminate large membrane vesicles, and then was further centrifuged at 100,000 rpm for 70 min to deposit exosomes. For subsequent experimental verification, exosomes were obtained using the ExoQuick solution (SBI, Fremont, CA, USA) performed according to the manufacturer’s instructions, with exosomes resuspended in elution buffer and stored at 80°C before further analysis.

### 4.3. Identification of Bovine Adipocyte Exosomes

To evaluate the exosome characteristics, nanoparticle tracking analysis (NTA) was performed using a Flow NanoAnalyzer (NanoFCM, Fujian, China) according to the manufacturer’s instructions. In addition, the morphology of exosome pellets was observed by transmission electron microscopy (TEM) HT-7800 (HITACHI, Tokyo, Japan), and exosomal surface markers (CD63 and Alix) were detected via Western blotting analysis. All samples were evaluated in triplicate.

### 4.4. RNA Extractions and Quantitation

Total RNA was extracted from bovine adipocytes and exosomes using the Trizol reagent (TAKARA, Shiga, Japan) and RA806TC-1 (SBI, Palo Alto, CA, USA) following manufacturer’s protocols. The RNA quantity and concentration were assessed using NanoDrop 2000 (Thermo Fisher Scientific, Waltham, MA, USA), and the integrity was determined by agarose gel electrophoresis. Total RNA samples were stored at −80 °C before further analysis.

### 4.5. RNA Library Construction and Deep Sequencing

The construction and deep sequencing of RNA libraries were accomplished with the assistance of Shenzhen BGI Co., Ltd. Briefly, RNA samples from bovine adipocytes (*n* = 3) and exosomes (*n* = 3) were ligated with 5′ and 3′ adapters, followed by cDNA synthesis using adaptor-specific primers. After PCR amplification, the RNA libraries were gel purified and the library quality was assessed. Then, sequencing was performed on the DNBSEQ/BGISEQ-500 platform (BGI, Shenzhen, China).

### 4.6. RNA Sequence Analysis

Clean reads were saved in FASTQ format, and low quality reads were removed from raw data using SOAPnuke (https://github.com/BGI-flexlab/SOAPnuke). Valid reads were aligned against both the bovine miRNA sequences (Release 21) and the reference bovine genome (GCF_000003205.7_Btau_5.0.1) to annotate the known RNAs using HISAT2 (http://www.ccb.jhu.edu/software/hisat) and Bowtie2 was used for miRNA. RSEM (http://deweylab.biostat.wisc.edu/rsem) was used to estimate RNA abundances, and fragments per kilobase of transcript per million mapped reads (FPKM) were determined for RNA transcript expression levels in this study. Here, FPKM > 0 was used as a value of cut-off limit for RNA detection across all samples.

### 4.7. Functional Enrichment Analysis

For the functional exploration of exosomal mRNAs, the top 500 abundant exosomal mRNAs were screened, and functional annotations including GO analysis, KEGG pathway analysis, and reactome enrichment analysis were performed using Phyper function in R software. Q-value < 0.05 (*p*-value < 0.05 for KEGG pathway analysis) was used as a threshold of significance.

### 4.8. Construction and Analysis of the Network of Exosomal RNAs

In our study, the 12 most abundant miRNA were chosen from 48 exosomal miRNA, and their target genes were further predicted using miRTarBase, an experimentally confirmed miRNA–mRNA interactions database (Chou et al., 2018). These acquired miRNA–mRNA pairs were visualized using Cytoscape software (http://www.cytoscape.org/) and GO and KEGG pathway analysis were performed for exosomal miRNA function annotation. Previous work has shown that lncRNAs can regulate gene expression by acting as miRNA sponges as part of the competing endogenous RNAs network. TargetScan (http://www.targetscan.org/), miRanda (http://www.microrna.org/microrna/home.do), and RNAhybrid (https://bibiserv.cebitec.uni-bielefeld.de/rnahybrid) were used to predict lncRNA–miRNA target relationships. The target miRNAs of exosomal lncRNAs were further screened as MRE < −36 standard, and miRNA–mRNA pairs were assessed by bioinformatics analysis using miRTarBase program [54]. Ultimately, the lncRNA–miRNA–mRNA network was reconstructed and visualized by Cytoscape software (Cytoscape 3), and GO and KEGG pathway analyses were performed again for exosomal lncRNA function annotation.

## 5. Conclusions

Here, for the first time, we isolated and characterized exosomes derived from bovine adipocytes, and identified mRNAs, lncRNAs, and miRNAs with the potential to regulate recipient cell phenotype and modulate multiple cellular pathways. We detected a notable enrichment of RNAs in exosomes compared to parental cells, and the differences in RNAs suggest a complex sorting mechanism of RNAs into exosomes. The results of this work provide a better understanding of exosomal RNAs in bovine adipocytes and should facilitate further study of bovine adipogenesis and exosomal RNAs.

## Supplementary Materials

Supplementary Materials can be found at https://www.mdpi.com/1422-0067/21/23/9313/s1. Figure S1: Western blot analysis of exosomes derived from bovine adipocytes. Adipocytes lysates and exosomes were blotted for CD63 and ALIX. Figure S2: Bioanalyzer profiles of the total RNAs of bovine adipocytes and released exosomes. Table S1: Quality statistics of filtered reads. Table S2: Quality statistics of filtered reads (small RNA). Table S3: Alignment clean reads to the reference genes. Table S4: smallRNA Classification. Table S5: smallRNA length distribution. Table S6: BP result of the exosomal mRNAs. Table S7: CC result of the exosomal mRNAs. Table S8: MF result of the exosomal mRNAs. Table S9: KEGG pathway result of the exosomal mRNAs. Table S10: Reactome Enrichment result of the exosomal mRNAs. Table S11: BP result of the miRNA-mRNA network. Table S12: CC result of the miRNA-mRNA network. Table S13: MF result of the miRNA-mRNA network. Table S14: KEGG pathway result of the miRNA-mRNA network. Table S15: BP result of the lncRNA-miRNA-mRNA network. Table S16: CC result of the the lncRNA-miRNA-mRNA network. Table S17: MF result of the lncRNA-miRNA-mRNA network. Table S18: KEGG result of the lncRNA-miRNA-mRNA network.

## Abbreviations

ncRNAs	long noncoding RNAs
miRNA	microRNA
EXO	exosome
WAT	white adipose tissue
NTA	nanoparticle tracking analysis
TEM	transmission electron microscopy
FPKM	fragments per kilobase of transcript per million mapped reads
GO	gene ontologies
KEGG	Kyoto Encyclopedia of Genes and Genomes
PCA	principle component analysis
BP	Biological Process
CC	Cellular Component
MF	Molecular Function
nSMase2	neural sphingomyelinase 2
hnRNP	heterogeneous nuclear ribonucleoproteins
YBX1	Y-box protein 1
RPS	ribosomal protein small subunit
RPL	ribosomal protein large subunit
ceRNA	competing endogenous RNA
MSCs	mesenchymal stromal cells

## References

[1] Kajimura S. (2017). Adipose tissue in 2016: Advances in the understanding of adipose tissue biology. Nat. Rev. Endocrinol..

[2] Torres S., Fabersani E., Marquez A., Gauffin-Cano P. (2019). Adipose tissue inflammation and metabolic syndrome. The proactive role of probiotics. Eur. J. Nutr..

[3] Scheja L., Heeren J. (2019). The endocrine function of adipose tissues in health and cardiometabolic disease. Nat. Rev. Endocrinol..

[4] Ghaben A.L., Scherer P.E. (2019). Adipogenesis and metabolic health. Nat. Rev. Mol. Cell Biol..

[5] Park J., Euhus D.M., Scherer P.E. (2011). Paracrine and endocrine effects of adipose tissue on cancer development and progression. Endocr. Rev..

[6] van Niel G., D’angelo G., Raposo G. (2018). Shedding light on the cell biology of extracellular vesicles. Nat. Rev. Mol. Cell Biol..

[7] Kalluri R., Lebleu V. (2020). The biology function and biomedical applications of exosomes. Science.

[8] Mcandrews K., Kalluri R. (2019). Mechanisms associated with biogenesis of exosomes in cancer. Mol. Cancer.

[9] He C., Zheng S., Luo Y., Wang B. (2018). Exosome Theranostics: Biology and Translational Medicine. Theranostics.

[10] Conigliaro A., Fontana S., Raimondo S., Alessandro R. (2017). Exosomes: Nanocarriers of Biological Messages. Adv. Exp. Med. Biol..

[11] Mori M.A., Ludwig R.G., Garcia-martin R., Brandão B.B., Kahn C.R. (2019). Extracellular miRNAs: From Biomarkers to Mediators of Physiology and Disease. Cell Metab..

[12] Li Y., Yin Z., Fan J., Zhang S., Yang W. (2019). The roles of exosomal miRNAs and lncRNAs in lung diseases. Signal Transduct. Target. Ther..

[13] Pan Y., Hui X., Hoo R.L.C., Ye D., Chan C.Y.C., Feng T., Wang Y., Lam K.S.L., Xu A. (2019). Adipocyte-secreted exosomal microRNA-34a inhibits M2 macrophage polarization to promote obesity-induced adipose inflammation. J. Clin. Investig..

[14] Lee B., Shao J. (2014). Adiponectin and energy homeostasis. Rev. Endocr. Metab. Dis..

[15] Berry R., Rodeheffer M.S. (2013). Characterization of the adipocyte cellular lineage in vivo. Nat. Cell Biol..

[16] Guimarães-camboa N., Evans S.M. (2017). Are Perivascular Adipocyte Progenitors Mural Cells or Adventitial Fibroblasts?. Cell Stem Cell.

[17] Lin R.Z., Moreno-luna R., Muñoz-hernandez R., Li D., Jaminet S.C., Greene A.K., Melero-martin J.M. (2013). Human white adipose tissue vasculature contains endothelial colony-forming cells with robust in vivo vasculogenic potential. Angiogenesis.

[18] Poglio S., De Toni F., Lewandowski D., Minot A., Arnaud E., Barroca V., Laharrague P., Casteilla L., Cousin B. (2012). In situ production of innate immune cells in murine white adipose tissue. Blood.

[19] Ribeiro-Rodrigues T., Martins-Marques T., Morel S., Kwak B.R., Girao H. (2017). Role of connexin 43 in different forms of intercellular communication—Gap junctions, extracellular vesicles and tunnelling nanotubes. J. Cell Sci..

[20] van den Boorn J.G., Schlee M., Coch C., Hartmann G. (2011). SiRNA delivery with exosome nanoparticles. Nat. Biotechnol..

[21] Batagov A.O., Kurochkin I.V. (2013). Exosomes secreted by human cells transport largely mRNA fragments that are enriched in the 3′-untranslated regions. Biol. Direct..

[22] Qu L., Ding J., Chen C., Wu Z.J., Liu B., Gao Y., Chen W., Liu F., Sun W., Li X.F. (2016). Exosome-Transmitted lncARSR Promotes Sunitinib Resistance in Renal Cancer by Acting as a Competing Endogenous RNA. Cancer Cell.

[23] Kosaka N., Iguchi H., Hagiwara K., Yoshioka Y., Takeshita F., Ochiya T. (2013). Neutral sphingomyelinase 2 (nSMase2)-dependent exosomal transfer of angiogenic microRNAs regulate cancer cell metastasis. J. Biol. Chem..

[24] Koppers-Lalic D., Hackenberg M., Bijnsdorp I.V., Van Eijndhoven M.A., Sadek P., Sie D., Zini N., Middeldorp J.M., Ylstra B., De Menezes R.X. (2014). Nontemplated nucleotide additions distinguish the small RNA composition in cells from exosomes. Cell Rep..

[25] Mckenzie A.J., Hoshino D., Hong N.H., Cha D.J., Franklin J.L., Coffey R.J., Patton J.G., Weaver A.M. (2016). KRAS-MEK Signaling Controls Ago2 Sorting into Exosomes. Cell Rep..

[26] Villarroya-beltri C., Gutiérrez-vázquez C., Sánchez-cabo F., Pérez-hernández D., Vázquez J., Martin-cofreces N., Martinez-herrera D.J., Pascual-montano A., Mittelbrunn M., Sánchez-madrid F. (2013). Sumoylated hnRNPA2B1 controls the sorting of miRNAs into exosomes through binding to specific motifs. Nat. Commun..

[27] Shurtleff M.J., Temoche-diaz M.M., Karfilis K.V., Ri S., Schekman R. (2016). Y-box protein 1 is required to sort microRNAs into exosomes in cells and in a cell-free reaction. Elife.

[28] Ahadi A., Brennan S., Kennedy P.J., Hutvagner G., Tran N. (2016). Long non-coding RNAs harboring miRNA seed regions are enriched in prostate cancer exosomes. Sci. Rep..

[29] Chen T., Xi Q.Y., Ye R.S., Cheng X., Qi Q.E., Wang S.B., Shu G., Wang L.N., Zhu X.T., Jiang Q.Y. (2014). Exploration of microRNAs in porcine milk exosomes. BMC Genom..

[30] Jenjaroenpun P., Kremenska Y., Nair V.M., Kremenskoy M., Joseph B., Kurochkin I.V. (2013). Characterization of RNA in exosomes secreted by human breast cancer cell lines using next-generation sequencing. Peerj.

[31] Wei Z., Batagov A.O., Schinelli S., Wang J., Wang Y., Elfatimy R., Rabinovsky R., Balaj L., Chen C.C., Hochberg F. (2017). Coding and noncoding landscape of extracellular RNA released by human glioma stem cells. Nat. Commun..

[32] Zhao K., Liang G., Sun X., Guan L.L. (2016). Comparative miRNAome analysis revealed different miRNA expression profiles in bovine sera and exosomes. BMC Genom..

[33] Xiao D., Ohlendorf J., Chen Y., Taylor D.D., Rai S.N., Waigel S., Zacharias W., Hao H., Mcmasters K.M. (2012). Identifying mRNA, microRNA and protein profiles of melanoma exosomes. PLoS ONE.

[34] Gregson A.L., Hoji A., Injean P., Poynter S.T., Briones C., Palchevskiy V., Weigt S.S., Shino M.Y., Derhovanessian A., Sayah D. (2015). Altered Exosomal RNA Profiles in Bronchoalveolar Lavage from Lung Transplants with Acute Rejection. Am. J. Respir. Crit. Care.

[35] Valadi H., Ekström K., Bossios A., Sjöstrand M., Lee J.J., Lötvall J.O. (2007). Exosome-mediated transfer of mRNAs and microRNAs is a novel mechanism of genetic exchange between cells. Nat. Cell Biol..

[36] Ratajczak J., Wysoczynski M., Hayek F., Janowska-Wieczorek A., Ratajczak M.Z. (2006). Membrane-derived microvesicles: Important and underappreciated mediators of cell-to-cell communication. Leukemia.

[37] Esau C., Kang X., Peralta E., Hanson E., Marcusson E.G., Ravichandran L.V., Sun Y., Koo S., Perera R.J., Jain R. (2004). MicroRNA-143 regulates adipocyte differentiation. J. Biol. Chem..

[38] Dong P., Mai Y., Zhang Z., Mi L., Wu G., Chu G., Yang G., Sun S. (2014). MiR-15a/b promote adipogenesis in porcine pre-adipocyte via repressing FoxO1. Acta Biochim. Biophys. Sin..

[39] Sun T., Fu M., Bookout A.L., Kliewer S.A., Mangelsdorf D.J. (2009). MicroRNA let-7 regulates 3T3-L1 adipogenesis. Mol. Endocrinol. (Baltim. Md.).

[40] Grosshans H., Johnson T., Reinert K.L., Gerstein M., Slack F.J. (2005). The temporal patterning microRNA let-7 regulates several transcription factors at the larval to adult transition in C. elegans. Dev. Cell.

[41] Yu Y., Du H., Wei S., Feng L., Li J., Yao F., Zhang M., Hatch G.M., Chen L. (2018). Adipocyte-Derived Exosomal MiR-27a Induces Insulin Resistance in Skeletal Muscle Through Repression of PPARγ. Theranostics.

[42] Liu Y., Tan J., Ou S., Chen J., Chen L. (2019). Adipose-derived exosomes deliver miR-23a/b to regulate tumor growth in hepatocellular cancer by targeting the VHL/HIF axis. J. Physiol. Biochem..

[43] Guduric-fuchs J., O’connor A., Camp B., O’neill C.L., Medina R.J., Simpson D.A. (2012). Selective extracellular vesicle-mediated export of an overlapping set of microRNAs from multiple cell types. BMC Genom..

[44] Yang J.S., Lai E.C. (2010). Dicer-independent, Ago2-mediated microRNA biogenesis in vertebrates. Cell Cycle (Georget. Tex.).

[45] Tay Y., Rinn J., Pandolfi P.P. (2014). The multilayered complexity of ceRNA crosstalk and competition. Nature.

[46] Li M., Sun X., Cai H., Sun Y., Plath M., Li C., Lan X., Lei C., Lin F., Bai Y. (2016). Long non-coding RNA ADNCR suppresses adipogenic differentiation by targeting miR-204. Biochim. Biophys. Acta.

[47] Li M., Xie Z., Wang P., Li J., Liu W., Tang S., Liu Z., Wu X., Wu Y., Shen H. (2018). The long noncoding RNA GAS5 negatively regulates the adipogenic differentiation of MSCs by modulating the miR-18a/CTGF axis as a ceRNA. Cell Death Dis..

[48] Zhang T., Liu H., Mao R., Yang H., Zhang Y., Zhang Y., Guo P., Zhan D., Xiang B., Liu Y. (2020). The lncRNA RP11-142A22.4 promotes adipogenesis by sponging miR-587 to modulate Wnt5β expression. Cell Death Dis..

[49] Ha D., Yang N., Nadithe V. (2016). Exosomes as therapeutic drug carriers and delivery vehicles across biological membranes: Current perspectives and future challenges. Acta Pharm. Sin. B.

[50] Ying W., Riopel M., Bandyopadhyay G., Dong Y., Birmingham A., Seo J.B., Ofrecio J.M., Wollam J., Hernandez-carretero A., Fu W. (2017). Adipose Tissue Macrophage-Derived Exosomal miRNAs Can Modulate In Vivo and In Vitro Insulin Sensitivity. Cell.

[51] Jin Y., Wang J., Li H., Gao S., Shi R., Yang D., Wang X., Wang X., Zhu L., Wang X. (2018). Extracellular Vesicles Secreted by Human Adipose-derived Stem Cells (hASCs) Improve Survival Rate of Rats with Acute Liver Failure by Releasing lncRNA H19. EBioMedicine.

[52] Hirai S., Matsumoto H., Moriya N.H., Kawachi H., Yano H. (2007). Follistatin rescues the inhibitory effect of activin A on the differentiation of bovine preadipocyte. Domest. Anim. Endocrinol..

[53] Lengi A.J., Corl B.A. (2010). Factors influencing the differentiation of bovine preadipocytes in vitro. J. Anim. Sci..

[54] Chou C.H., Shrestha S., Yang C.D., Chang N.W., Lin Y.L., Liao K.W., Huang W.C., Sun T.H., Tu S.J., Lee W.H. (2018). miRTarBase update 2018: A resource for experimentally validated microRNA-target interactions. Nucleic Acids Res..

