# Supplementary figures and images for: Characterization and Transcriptome Analysis of Exosomal and Nonexosomal RNAs in Bovine Adipocytes
Source: Int J Mol Sci. 2020 Dec 7;21(23):9313. doi: 10.3390/ijms21239313 (PMC7730049; doi:10.3390/ijms21239313)

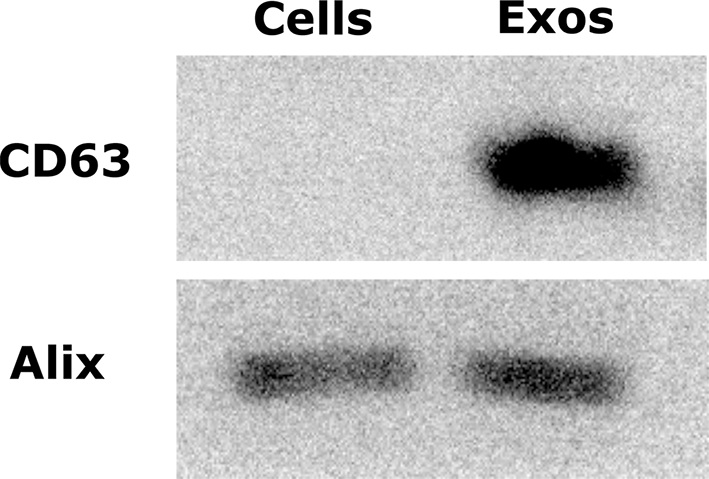

Supplement: Supplementary file 1 [file ijms-21-09313-s001.zip › suppl/Figure S1.tif]

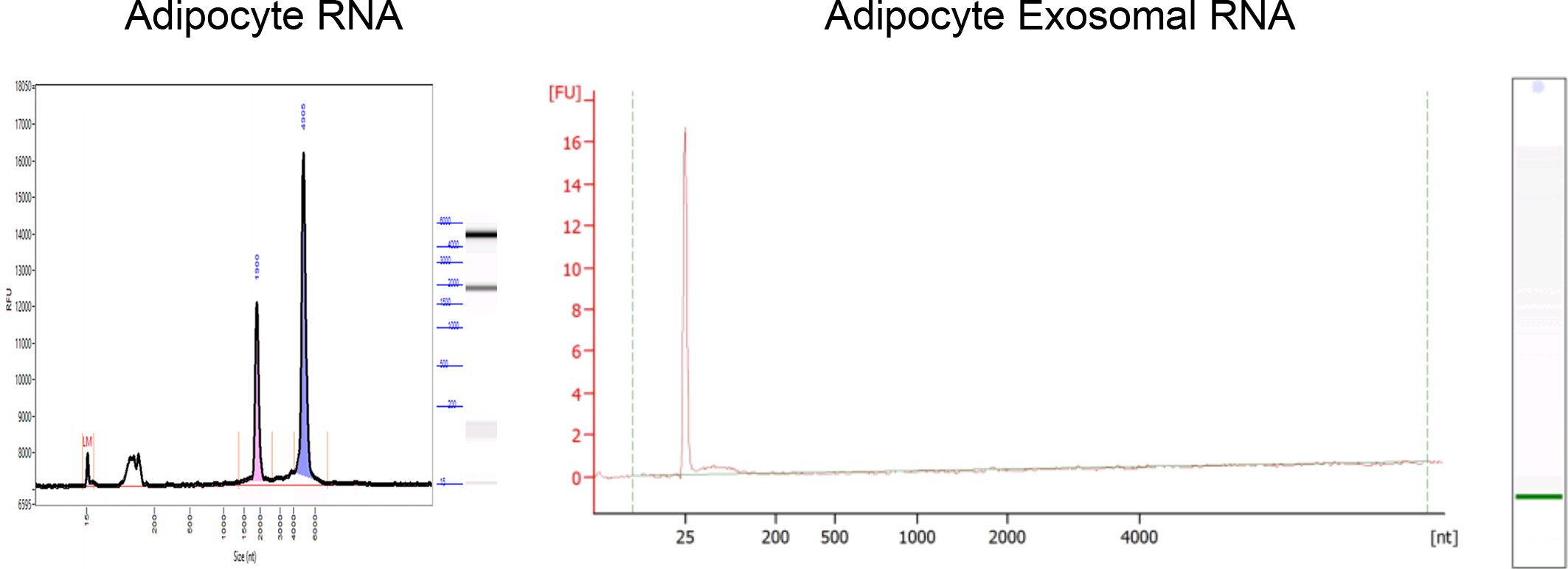

Supplement: Supplementary file 1 [file ijms-21-09313-s001.zip › suppl/Figure S2.tif]
